# Optimizing pediatric shock wave lithotripsy: stepwise energy escalation vs. conventional fixed energy

**DOI:** 10.3389/fped.2026.1736104

**Published:** 2026-03-30

**Authors:** Mehmet Ali Ergül, İbrahim Üntan, Sultan Üntan, Ayşe Betül Ergül, Deniz Demirci

**Affiliations:** 1 Department of Urology, Doha Clinic Hospital, Doha, Qatar; 2 Department of Urology, Ahi Evran University School of Medicine, Kırşehir, Türkiye; 3 Department of Family Medicine, Atatürk Sanatoryum Education and Research Hospital, Ankara, Türkiye; 4 Department of Pediatrics, Muaither Health Center, Primary Health Care Corporation, Doha, Qatar; 5 Department of Urology, Erciyes University School of Medicine, Kayseri, Türkiye

**Keywords:** extracorporeal shock wave lithotripsy, pediatric urolithiasis, stepwise energy escalation, stone-free rate, urinary calculi

## Abstract

**Background:**

Pediatric extracorporeal shock wave lithotripsy (ESWL) is widely used, but the optimal energy-delivery strategy remains uncertain. Adult data suggest that stepwise energy escalation may enhance fragmentation and limit tissue injury; pediatric evidence is limited.

**Methods:**

We conducted a single-center retrospective cohort of 81 children treated with ESWL using either a stepwise energy protocol (*n* = 41) or a conventional fixed-energy protocol (*n* = 40). The stepwise protocol began at 10 kV with 1 kV increases every 250 shocks up to 13 kV (maximum 3,000 shocks per session); the conventional protocol used a fixed 13 kV. Primary outcome was 3-month stone-free status, defined as no visible stones on follow-up imaging, excluding fragments ≤3 mm. Secondary outcomes included stone-free status after the first session, number of sessions, auxiliary procedures, and complications.

**Results:**

After the first session, stone-free status was observed in 73.2% (30/41) with the stepwise protocol vs. 55.0% (22/40) with the conventional protocol. At 3 months, rates were 95.1% (39/41) vs. 87.5% (35/40). When fragments ≤3 mm were considered clearance, overall rates were 97.6% (40/41) vs. 95.0% (38/40). More children achieved clearance in a single session with the stepwise protocol (30 vs. 22). Auxiliary ureteroscopy was required in 1 vs. 2 cases. Only minor, self-limited events (hematuria, transient pain/colic) were reported.

**Conclusion:**

Stepwise energy escalation was associated with numerically higher clearance and fewer sessions than fixed-energy ESWL, without added morbidity. Prospective multicenter studies are needed to confirm these findings.

## Introduction

Pediatric urolithiasis is increasingly recognized worldwide, with rising incidence attributed to dietary patterns, metabolic risk, and broader imaging use (1, 2). When intervention is required, extracorporeal shock wave lithotripsy (ESWL) is widely used for appropriately selected stones because it is noninvasive, repeatable, and generally safe in children (3).

Despite its broad adoption, the optimal energy-delivery strategy for ESWL remains uncertain (4). In routine practice, some centers apply a fixed high energy throughout the session, while others use a stepwise escalation protocol designed to initiate fragmentation gently and then increase energy in a controlled manner (5).

Adult studies suggest that stepwise energy escalation can enhance fragmentation efficiency and reduce tissue injury compared with fixed-energy approaches (6). However, pediatric physiology, stone composition, and anesthesia considerations differ from adults, and children are particularly sensitive to session number and cumulative exposure (7, 8).

Evidence directly comparing stepwise vs. conventional fixed-energy ESWL in children is sparse, leaving a practical gap for clinicians who must choose a protocol in everyday care (9, 10).

To address this gap, we evaluated outcomes of stepwise vs. conventional fixed-energy ESWL in a single-center pediatric cohort. Our primary objective was to compare stone-free status at 3 months; secondary objectives included stone-free status after the first session, the number of sessions required for clearance, the need for auxiliary procedures, and complications. We hypothesized that stepwise energy escalation would be associated with at least comparable safety and improved treatment efficiency relative to a conventional fixed-energy protocol.

## Materials and methods

### Study design and setting

This was a single-center retrospective comparative cohort of consecutive pediatric patients undergoing ESWL between 2008 and 2017, conducted under institutional ethics approval. Reporting adheres to the STROBE guideline (11).

### Participants and eligibility

All children (<18 years) treated with ESWL at our institution during the study period were screened. Patients were included if they underwent ESWL for renal or ureteral stones meeting standard institutional criteria: stone size ≤20 mm, absence of active urinary tract infection, no uncorrectable urinary obstruction distal to the stone, and no bleeding diathesis. Stones in the renal pelvis, upper and middle calices, and proximal ureter were considered suitable candidates; lower-pole stones were evaluated individually based on size and infundibulopelvic anatomy. All cases were reviewed at the biweekly multidisciplinary council meeting—convening pediatric nephrology, pediatric surgery, and urology—before treatment allocation (3, 9, 12). Standard exclusions were applied: active urinary tract infection, uncorrected urinary obstruction, bleeding diathesis or anticoagulation that could not be safely interrupted, pregnancy, and inability to complete follow-up imaging. Consecutive eligible cases were assigned to one of two contemporaneous treatment protocols (stepwise vs. conventional) according to routine practice at the time.

### Interventions

Stone localization was performed in accordance with the ALARA principle: ultrasonography was used as the primary modality throughout the procedure for real-time, radiation-free monitoring. Fluoroscopy was applied only at the final step for definitive localization of radiopaque stones, using minimal pulsed low-dose exposure. This final fluoroscopic image also allowed procedural documentation via the Dornier Compact Delta's integrated interface with the institutional PACS system. Radiolucent stones were localized exclusively by ultrasonography (8, 13). Procedures were performed on a Dornier Compact Delta lithotripter (electromagnetic source) equipped with integrated fluoroscopy and ultrasonography capabilities for stone localization (13). The decision for a repeat ESWL session was made based on imaging assessment performed at the 15-day follow-up visit. Patients with residual stones larger than 3 mm on plain radiography or ultrasonography were offered a repeat session. A minimum interval of two weeks between sessions was observed to allow resolution of any post-treatment renal edema or hematoma before retreatment (12, 14).

In the stepwise protocol, energy was initiated at 10 kV and increased by 1 kV every 250 shocks up to a maximum of 13 kV, with a maximum of 3,000 shocks per session. In the conventional protocol, a fixed energy of 13 kV was applied throughout the session, with the same maximum of 3,000 shocks.

All children were managed under institutional pediatric anesthesia and monitoring standards, with intravenous sedation/analgesia administered at the discretion of the anesthesiologist, along with routine hydration. Coupling quality was checked throughout the procedure, and re-treatment sessions were scheduled based on clinical and imaging review.

### Follow-up and imaging

Patients were reviewed at least every 15 days, consistent with institutional protocol to allow timely detection of residual fragments and guide retreatment decisions. Stone status was assessed in accordance with the ALARA principle: ultrasonography was used as the primary follow-up imaging modality for all patients. Plain radiography was reserved for radiopaque stones only when ultrasonographic assessment was inconclusive, thereby minimizing cumulative radiation exposure during the follow-up period. Definitive outcome assessment was performed at 3 months after the index ESWL session or after the final session when multiple sessions were required.

### Outcomes and definitions

The primary outcome was the three-month stone-free rate (SFR), defined as the absence of visible stones on follow-up imaging, excluding residual fragments of ≤3 mm. The study endpoint was three months from the index ESWL session; patients requiring multiple sessions completed all retreatment within this window.

Secondary outcomes included stone-free status after the first session, the number of sessions required to achieve clearance (one, two, or three), the need for auxiliary procedures, and the occurrence of complications within 30 days. Definitive clearance was defined as complete clearance or residual fragments of ≤3 mm (CIRF).

Complications within 30 days of each ESWL session were extracted from the medical record and summarized as *n* (%). Between-group comparisons of complication rates were performed using Fisher's exact test.

### Bias and confounding control

Because allocation reflected routine practice rather than randomization, potential confounding by baseline differences was considered *a priori*. We compared baseline characteristics between groups and present outcomes with absolute counts and percentages. Given the sample size, adjusted analyses were exploratory only.

### Statistical analysis

Continuous variables are summarized as medians with ranges (sessions per patient are reported as means); categorical variables as counts and percentages. Between-group comparisons used the Mann–Whitney U test for continuous variables and Fisher's exact test for categorical variables. For key binary outcomes, effect sizes are presented as risk ratios (RRs) with 95% confidence intervals calculated by the log (Wald) method; single-group confidence intervals for proportions (figures) use the Wilson score method. Two-sided *p* values <0.05 were considered statistically significant. Analyses were performed with standard statistical software IBM SPSS Statistics for Windows, Version 24.0 (IBM Corp., Armonk, NY). (Final *p* values and confidence intervals are reported in the Results where applicable.)

## Results

### Participants and baseline

Eighty-one children were included (stepwise *n* = 41, conventional *n* = 40) with no exclusions or losses to follow-up. Baseline characteristics were similar between groups (Table 1). The stepwise group comprised 20/21 F/M (median age 7.7 years, range 1–15) and the conventional group 13/27 F/M (median age 5.5 years, range 1.25–16). Median stone size was 0.87 cm (range 0.3–1.7) vs. 1.03 cm (range 0.5–1.5) in stepwise and conventional groups, respectively (Table 1).

**Table 1:** Baseline characteristics of children undergoing stepwise vs. conventional ESWL.

Variable	Stepwise (*n* = 41)	Conventional (*n* = 40)	*p*
Female sex, *n* (%)	20 (48.8)	13 (32.5)	0.16*
Age, years, median (range)	7.7 (1–15)	5.5 (1.25–16)	0.07†
Stone size, cm, median [range]	0.87 (0.3–1.7)	1.03 (0.5–1.5)	0.21†

Values are median (range) or *n* (%). *p*-values by:

* Fisher's exact test (categorical) and

† Mann–Whitney *U* test (continuous). ESWL, extracorporeal shock wave lithotripsy. Stone location, number, and primary/secondary status were not systematically recorded in the source dataset and could not be compared between groups; the minimum stone size of 0.3 cm in the stepwise group reflects an individual clinical decision made at the multidisciplinary council.

### Primary outcome

**Figure 1:**
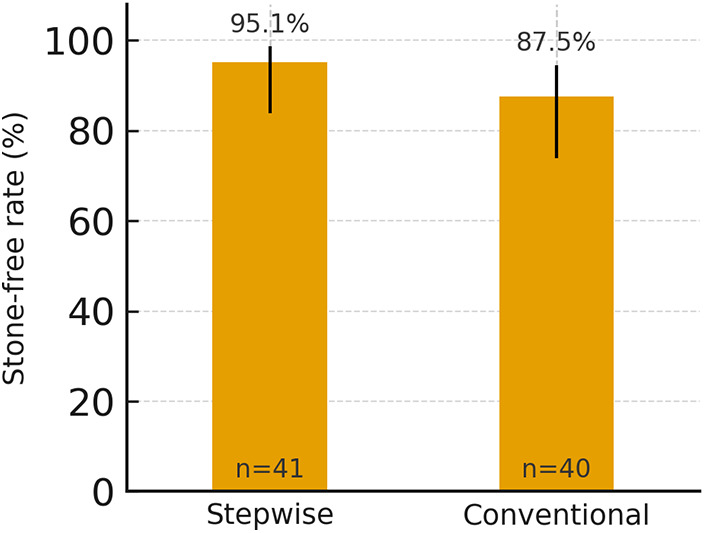
Stone-free rate at 3 months in stepwise and conventional groups with 95% Wilson confidence intervals. Percentages are shown above bars; *n* indicates group size. *ESWL, extracorporeal shock wave lithotripsy.*

The primary outcome, three-month stone-free rate, was achieved in 39 of 41 children (95.1%) in the stepwise group compared with 35 of 40 (87.5%) in the conventional group (RR 1.09, 95% CI 0.96–1.23; *p* = 0.264) (Figure 1; Table 2).

**Table 2:** Stone-free outcomes at first session and at 3 months, and definitive clearance including CIRF ≤3 mm.

Outcome	Stepwise (*n* = 41)	Conventional (*n* = 40)	*p*
Stone-free after 1st session, *n* (%)	30 (73.2)	22 (55.0)	0.108
Stone-free at 3 months, *n* (%)	39 (95.1)	35 (87.5)	0.264
Definitive clearance incl. CIRF ≤3 mm, *n* (%)	40 (97.6)	38 (95.0)	0.616
Auxiliary ureteroscopy, *n* (%)	1 (2.4)	2 (5.0)	1.000

Values are *n* (%); *p*-values by Fisher's exact test. SFR, stone-free rate; CIRF, clinically insignificant residual fragments (≤3 mm).

### Secondary outcomes

**Figure 2:**
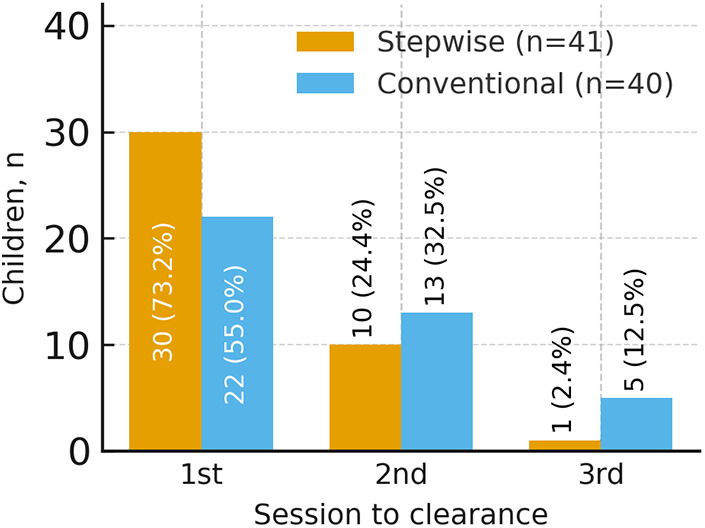
Sessions required for clearance in stepwise (*n* = 41) and conventional (*n* = 40) groups. Bars display the number of children; within-group percentages are provided in Table 3. *ESWL, extracorporeal shock wave lithotripsy.*

**Figure 3:**
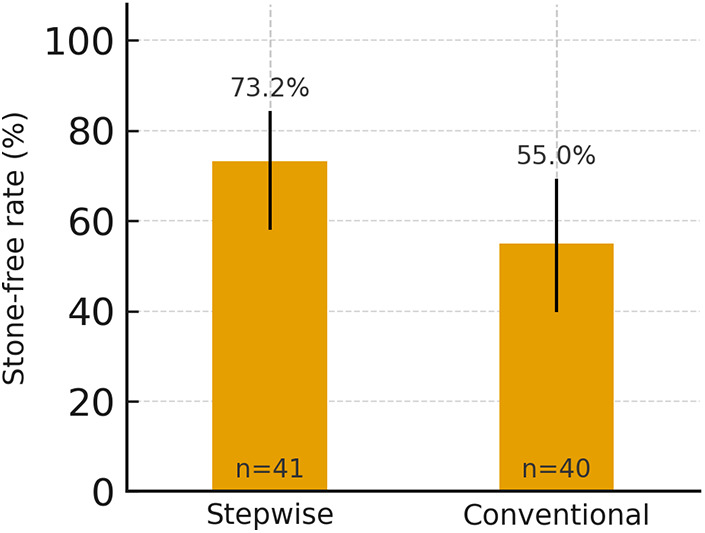
Stone-free rate after the first ESWL session in stepwise and conventional groups with 95% Wilson confidence intervals. Percentages are shown above bars; *n* indicates group size. *ESWL, extracorporeal shock wave lithotripsy.*

**Figure 4:**
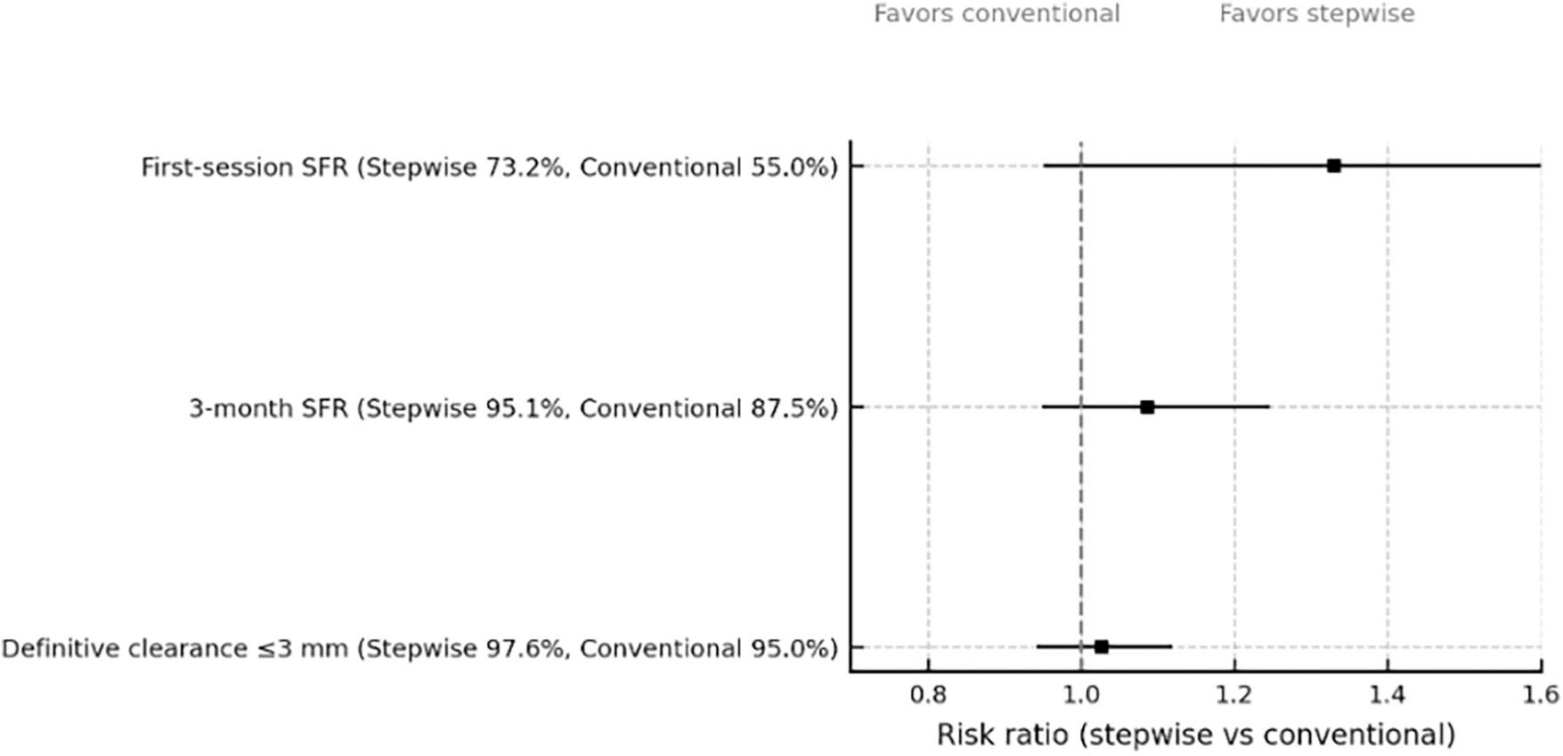
Risk ratios (RR) for stone-free outcomes comparing stepwise vs. conventional ESWL with 95% confidence intervals; the vertical dashed line denotes no difference (RR = 1). *RR, risk ratio; ESWL, extracorporeal shock wave lithotripsy.*

The distribution of sessions required for clearance was 30/10/1 (one, two, or three sessions) in the stepwise group and 22/13/5 in the conventional group (Figure 2; Table 3). Mean sessions per patient were 1.29 and 1.58, respectively, indicating that a greater proportion of children in the stepwise group cleared in a single session (30/41 vs. 22/40) (Figure 3). Between-group risk ratios with 95% confidence intervals are summarized in Figure 4.

**Table 3:** Sessions required for clearance and need for auxiliary ureteroscopy.

Parameter	Stepwise (*n* = 41)	Conventional (*n* = 40)	*p*
Sessions to clearance, 1st, *n* (%)	30 (73.2)	22 (55.0)	0.108
Sessions to clearance, 2nd, *n* (%)	10 (24.4)	13 (32.5)	0.610
Sessions to clearance, 3rd, *n* (%)	1 (2.4)	5 (12.5)	0.200
Sessions per patient, mean	1.29	1.58	0.620

Values are *n* (%) unless indicated. Percentages are within-group. *p*-values by Fisher's exact test (rows 1–3) and Mann–Whitney U (mean sessions). Stepwise protocol: 10 → 13 kV (+1 kV/250 shocks; maximum 3,000 shocks/session). Conventional protocol: fixed 13 kV (maximum 3,000 shocks/session). ESWL, extracorporeal shock wave lithotripsy.

**Figure 5:**
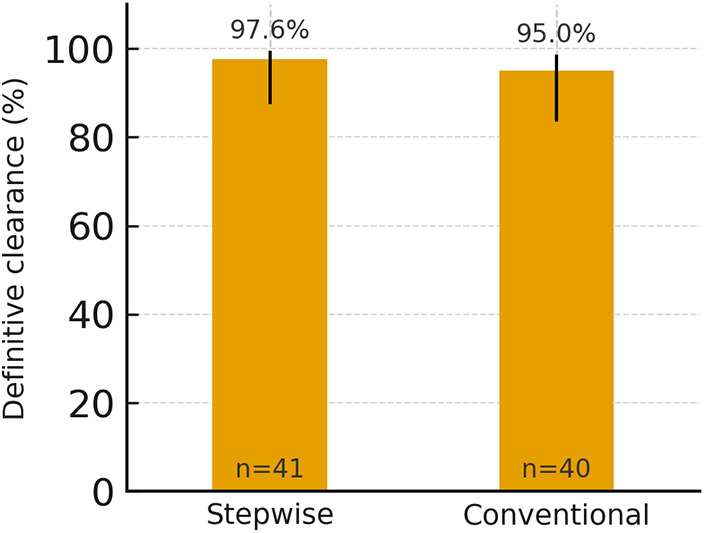
Definitive clearance including CIRF ≤3 mm in stepwise and conventional groups with 95% Wilson confidence intervals. Percentages are shown above bars; *n* indicates group size. *CIRF, clinically insignificant residual fragments; ESWL, extracorporeal shock wave lithotripsy*.

When clinically insignificant residual fragments (CIRF) ≤ 3 mm were considered clearance, definitive clearance rates were 40/41 (97.6%) vs. 38/40 (95.0%) (Figure 5; Table 2).

Minor, expected post-ESWL events were common and similar between groups, including hematuria (87.8% vs. 85.0%) and transient colic (80.5% vs. 77.5%; both *p* > 0.7). No major complications, such as febrile urinary tract infection, Steinstrasse, emergency visits, or hospitalization, occurred in either group (Table 4). One auxiliary ureteroscopy was required in the stepwise group and two in the conventional group (Table 3).

**Table 4:** Post-ESWL events within 30 days (minor expected effects).

Event	Stepwise (*n* = 41), *n* (%)	Conventional (*n* = 40), *n* (%)	*p*
Hematuria (self-limited)	36 (87.8)	34 (85.0)	0.791
Pain/colic (transient)	33 (80.5)	31 (77.5)	0.808

ESWL, extracorporeal shock wave lithotripsy. Minor, expected post-ESWL effects were common and comparable between groups. No major complications (febrile UTI, Steinstrasse, emergency visit, hospitalization) occurred in either group. *p*-values by Fisher's exact test.

## Discussion

In this single-center cohort of children undergoing ESWL, a stepwise energy-escalation protocol was associated with higher stone-free rates and fewer treatment sessions than a conventional fixed-energy protocol, without an apparent increase in morbidity. The direction and magnitude of effect were consistent across early (after the first session) and definitive (3-month) assessments, and the signal remained when clearance included clinically insignificant residual fragments ≤3 mm. Although group differences did not reach conventional levels of statistical significance in this sample, the pattern of benefit was coherent and clinically meaningful—particularly the greater proportion of children cleared after a single session.

### Interpretation and potential mechanisms

A gradual rise in energy may promote controlled nucleation and crack propagation while limiting early tissue stress, thereby improving fragmentation efficiency (15, 16). In children, who have smaller body habitus and may require sedation or anesthesia, an approach that maximizes early fragmentation can translate into fewer repeat sessions, less cumulative exposure to anesthesia, and reduced burden on families and services (17). The comparable safety profile observed here suggests that stepwise escalation does not trade efficacy for complications. These minor effects are expected after ESWL and were not clinically significant; importantly, no major complications were observed in either protocol.

### Clinical relevance

From a pragmatic standpoint, the higher first-session clearance with stepwise energy escalation can shorten the care pathway, reduce imaging and clinic revisits, and limit ancillary procedures (12, 18). This aligns with North American guidance on modality selection and technical conduct of ESWL in children, including attention to session minimization and anesthesia exposure (19). Even modest relative gains are meaningful in pediatrics, where each additional session implies logistics for guardians, school absence, and repeated peri-procedural care (20, 21). Centers that already perform ESWL could adopt a standardized stepwise protocol with minimal change in equipment or staffing.

### Context within existing literature

Adult series have suggested that stepwise (ramped) energy delivery enhances fragmentation and may reduce tissue injury compared with fixed-energy delivery (22). Pediatric reports are fewer and have largely emphasized modality choice (ESWL vs. ureteroscopy or percutaneous approaches) rather than protocol optimization within ESWL (23, 24). By focusing on how energy is delivered, this study adds practical evidence to an area where pediatric data remain limited (25, 26).

### Sensitivity and robustness

Outcomes were consistent whether clearance was defined strictly as stone-free or inclusively with residual fragments ≤3 mm. Session distribution also favored the stepwise protocol (more one-session clearances, fewer third sessions). These converging findings support the robustness of the observed pattern. It should be noted that the stepwise group had a somewhat smaller median stone size (0.87 vs. 1.03 cm), which may have contributed to the higher single-session clearance rate observed in that group. This imbalance, inherent to the retrospective non-randomized design, limits causal interpretation and is acknowledged as a limitation.

## Limitations

Several limitations merit emphasis. First, the retrospective design is subject to selection bias and unmeasured confounding; allocation reflected routine practice rather than randomization. Second, this was a single-center study, which may limit generalizability. Third, although baseline characteristics were broadly similar, minor imbalances (for example, stone size) could bias effect estimates. Fourth, complications were extracted from records without systematic adjudication, limiting granular safety comparisons. Fifth, follow-up centered on 3-month imaging; longer-term recurrence and functional outcomes were beyond scope. Finally, the study was not powered *a priori*; no prior pediatric data on stepwise vs. conventional ESWL were available to inform a formal sample size calculation at the time of design. *Post-hoc* power analysis revealed 22.9% power to detect the observed difference in the primary outcome (3-month SFR: 95.1% vs. 87.5%; Cohen's *h* = 0.28), with an estimated 214 patients per group required to achieve 80% power for this effect size. The absence of *a priori* power calculation is, however, not uncommon in single-center retrospective pediatric ESWL series, where patient numbers are inherently limited by disease prevalence. Additionally, stone location, number, and primary/secondary status were not available in the source dataset and could not be compared between groups; these variables may influence stone-free rates and represent an important limitation of this retrospective analysis.

### Strengths

Strengths include a clearly defined pediatric cohort treated with standardized protocols on the same platform, prespecified and clinically relevant endpoints, and consistent imaging-based follow-up. Reporting aligns with STROBE, and results are presented both as absolute counts and effect estimates to aid interpretation.

### Implications and next steps

In line with contemporary European guidance, standardized reporting of ESWL parameters and protocolization of energy ramping may improve comparability and quality of care (12). These data support stepwise energy escalation as a reasonable default for pediatric ESWL where equipment and expertise are available (27, 28). Future research should include prospective, preferably multicenter, comparative studies with standardized definitions of clearance (and an *a priori* threshold, such as 3 mm), uniform complication grading, and patient-centered outcomes (pain, school absence, quality of life); similar protocol heterogeneity has limited evidence synthesis in focused-ESWT for low-back pain (29, 30). Cost-effectiveness, radiation exposure, and anesthesia burden are additional priorities. A randomized design would best address residual confounding and clarify which children benefit most (e.g., by stone size, location, or composition).

## Conclusion

In this retrospective cohort, stepwise energy escalation was associated with numerically higher stone clearance and fewer treatment sessions compared with conventional fixed-energy ESWL, with similar safety. However, none of the between-group differences reached statistical significance, and the study was underpowered to detect small effect sizes. These findings are hypothesis-generating and should be interpreted accordingly. Prospective multicenter studies with adequate sample size are needed to confirm whether stepwise escalation confers a meaningful clinical advantage in children.

## Data Availability

The raw data supporting the conclusions of this article will be made available by the authors, without undue reservation.

## References

[1] Chen S Ma X Guo L Wang S Wu J Wu L The global, regional, and national burden of pediatric stone disease: 1990–2021 and projections for the next two decades. Front Pediatr. (2025) 13:1529407. 10.3389/fped.2025.1529407 40201663 PMC11975870

[2] Önen A. Urinary system stone disease in children. Turk J Pediatr Surg. (2013) 27:8–32. 10.5222/JTAPS.2013.008

[3] Burgos Lucena L Fernández Bautista B Parente Hernández A Ortiz Rodríguez R Angulo Madero JM. Extracorporeal shock wave lithotripsy and combined therapy in children: efficacy and long-term results. Front Pediatr. (2021) 9:609664. 10.3389/fped.2021.609664 34055678 PMC8155519

[4] Xiao K Zhou L Zhu S Lin L Di X Li H. Which frequency is better for pediatric shock wave lithotripsy? Low intermediate or high: a systematic review and meta-analysis. Front Surg. (2023) 10:1063159. 10.3389/fsurg.2023.1063159 37009606 PMC10050731

[5] Peng T Zhong H Hu B Zhao S. Minimally invasive surgery for pediatric renal and ureteric stones: a therapeutic update. Front Pediatr. (2022) 10:902573. 10.3389/fped.2022.902573 36061394 PMC9433542

[6] Lambert EH Walsh R Moreno MW Gupta M. Effect of escalating versus fixed voltage treatment on stone comminution and renal injury during extracorporeal shock wave lithotripsy: a prospective randomized trial. J Urol. (2010) 183(2):580–4. 10.1016/j.juro.2009.10.025 20018316

[7] Paraboschi I Gnech M De Marco EA Minoli DG Bebi C Zanetti SP Pediatric urolithiasis: current surgical strategies and future perspectives. Front Pediatr. (2022) 10:886425. 10.3389/fped.2022.886425 35757114 PMC9218273

[8] Halinski A Steyaert H Wojciech M Sobolewski B Haliński A. Endourology methods in pediatric population for kidney stones located in lower Calyx: Flexurs vs. micro Pcnl (microperc®). Front Pediatr. (2021) 9:640995. 10.3389/fped.2021.640995 34095024 PMC8175969

[9] Sultan S Aba Umer S Ahmed B Naqvi SAA Rizvi SAH. Update on surgical management of pediatric urolithiasis. Front Pediatr. (2019) 7:252. 10.3389/fped.2019.00252 31334207 PMC6616131

[10] Destro F Selvaggio GGO Lima M Riccipetitoni G Klersy C Di Salvo N Minimally invasive approaches in pediatric urolithiasis. The experience of two Italian centers of pediatric surgery. Front Pediatr. (2020) 8:377. 10.3389/fped.2020.00377 32793523 PMC7393988

[11] von Elm E Altman DG Egger M Pocock SJ Gøtzsche PC Vandenbroucke JP. The strengthening the reporting of observational studies in epidemiology (strobe) statement: guidelines for reporting observational studies. J Clin Epidemiol. (2008) 61(4):344–9. 10.1016/j.jclinepi.2007.11.008 18313558

[12] Türk C Petřík A Sarica K Seitz C Skolarikos A Straub M Eau guidelines on interventional treatment for urolithiasis. Eur Urol. (2016) 69(3):475–82. 10.1016/j.eururo.2015.07.041 26344917

[13] Vinit N Khoury A Lopez P Heidet L Botto N Traxer O Extracorporeal shockwave lithotripsy for cystine stones in children: an observational, retrospective, single-center analysis. Front Pediatr. (2021) 9:763317. 10.3389/fped.2021.763317 34869121 PMC8636798

[14] Zang M Dong Y Wang X Han C Jia J. New advances in efficacy prediction of extracorporeal shock wave lithotripsy in pediatrics: a narrative review. Front Pediatr. (2026) 13:1681384. 10.3389/fped.2025.1681384 41648050 PMC12868161

[15] McClain PD Lange JN Assimos DG. Optimizing shock wave lithotripsy: a comprehensive review. Rev Urol. (2013) 15(2):49–60. https://www.ncbi.nlm.nih.gov/pubmed/24082843 PMID: 24082843 24082843 PMC3784968

[16] Li X Liu Y. Focused ultrasound in modern medicine: bioengineering interfaces, molecular effects, and clinical breakthroughs. Front Bioeng Biotechnol. (2025) 13:1610846. 10.3389/fbioe.2025.1610846 40948965 PMC12426121

[17] Juliebø-Jones P Keller EX Tzelves L Beisland C Somani BK Gjengstø P Paediatric kidney stone surgery: state-of-the-art review. Ther Adv Urol. (2023) 15:17562872231159541. 10.1177/17562872231159541 36950219 PMC10026105

[18] Bultitude M Thomas K. Gently does it: ramping is the key to safe and efficient lithotripsy. Eur Urol. (2016) 69(2):274–5. 10.1016/j.eururo.2015.07.030 26227862

[19] Assimos D Krambeck A Miller NL Monga M Murad MH Nelson CP Surgical management of stones: American urological association/endourological society guideline, part I. J Urol. (2016) 196(4):1153–60. 10.1016/j.juro.2016.05.090 27238616

[20] McAdams S Shukla AR. Pediatric extracorporeal shock wave lithotripsy: predicting successful outcomes. Indian J Urol. (2010) 26(4):544–8. 10.4103/0970-1591.74457 21369388 PMC3034064

[21] Schwaderer AL Raina R Khare A Safadi F Moe SM Kusumi K. Comparison of risk factors for pediatric kidney stone formation: the effects of sex. Front Pediatr. (2019) 7:32. 10.3389/fped.2019.00032 30809514 PMC6379338

[22] Skuginna V Nguyen DP Seiler R Kiss B Thalmann GN Roth B. Does stepwise voltage ramping protect the kidney from injury during extracorporeal shockwave lithotripsy? Results of a prospective randomized trial. Eur Urol. (2016) 69(2):267–73. 10.1016/j.eururo.2015.06.017 26119561

[23] Harmon EP Neal DE Thomas R. Pediatric urolithiasis: review of research and current management. Pediatr Nephrol. (1994) 8(4):508–12. 10.1007/BF00856552 7947050

[24] Yuan Y Liang YN Li KF Ho YR Wu QL Zhao Z. A meta-analysis: retrograde intrarenal surgery vs. Percutaneous nephrolithotomy in children. Front Pediatr. (2023) 11:1086345. 10.3389/fped.2023.1086345 37205217 PMC10185757

[25] Nice guideline—renal and ureteric stones: assessment and management: nice (2019) renal and ureteric stones: assessment and management. BJU Int. (2019) 123(2):220–32. 10.1111/bju.14654 30656839

[26] Zhang ZG Lin QC Zhou QY Xu NF Zheng DQ Pan QZ Trend analysis of pediatric urolithiasis prevalence from 1990 to 2021 in the brics. Front Pediatr. (2025) 13:1551046. 10.3389/fped.2025.1551046 40061424 PMC11885269

[27] Lee SM Collin N Wiseman H Philip J. Optimisation of shock wave lithotripsy: a systematic review of technical aspects to improve outcomes. Transl Androl Urol. (2019) 8(Suppl 4):S389–97. 10.21037/tau.2019.06.07 31656745 PMC6790411

[28] Lv G Qi W Gao H Zhou Y Zhong M Wang K Safety and efficacy of extracorporeal shock wave lithotripsy vs. flexible ureteroscopy in the treatment of urinary calculi: a systematic review and meta-analysis. Front Surg. (2022) 9:925481. 10.3389/fsurg.2022.925481 36420414 PMC9676362

[29] Ferdinandov D. Effectiveness of extracorporeal shockwave therapy in chronic low back pain: a systematic review and meta-analysis. Front Med (Lausanne). (2024) 11:1435504. 10.3389/fmed.2024.1435504 39267973 PMC11390445

[30] Üntan İ Üntan S Tosun H Demirci D. Metabolic risk factors and the role of prophylaxis in pediatric urolithiasis. J Pediatr Urol. (2021) 17(2):215.e1–6. 10.1016/j.jpurol.2020.12.003 33342680

[31] Ergül MA. Ürolityazisli Çocuklarda adım adım ve konvansiyonel Şok dalga litotripsinin karşılaştırılması (Dissertation). Erciyes University, Department of Urology, Kayseri (2009).

